# *LOC550643*, a Long Non-coding RNA, Acts as Novel Oncogene in Regulating Breast Cancer Growth and Metastasis

**DOI:** 10.3389/fcell.2021.695632

**Published:** 2021-07-20

**Authors:** Kuo-Wang Tsai, Kian-Hwee Chong, Chao-Hsu Li, Ya-Ting Tu, Yi-Ru Chen, Ming-Cheng Lee, Shih-Hsuan Chan, Lu-Hai Wang, Yao-Jen Chang

**Affiliations:** ^1^Department of Research, Taipei Tzu Chi Hospital, Buddhist Tzu Chi Medical Foundation, New Taipei City, Taiwan; ^2^Division of General Surgery, Department of Surgery, Taipei Tzu Chi Hospital, Buddhist Tzu Chi Medical Foundation, New Taipei City, Taiwan; ^3^Graduate Institute of Clinical Medicine, College of Medicine, National Taiwan University, Taipei, Taiwan; ^4^Graduate Institute of Integrated Medicine, China Medical University, Taichung, Taiwan; ^5^Chinese Medicine Research Center, China Medical University, Taichung, Taiwan; ^6^Institute of Molecular Medicine, College of Life Science, National Tsing Hua University, Hsinchu, Taiwan; ^7^School of Medicine, Tzu Chi University, Hualien, Taiwan

**Keywords:** breast cancer, ncRNA, metastasis, lncRNA, microRNA

## Abstract

Metastatic disease is responsible for over 90% of death in patients with breast cancer. Therefore, identifying the molecular mechanisms that regulate metastasis and developing useful therapies are crucial tasks. Long non-coding RNAs (lncRNAs), which are non-coding transcripts with >200 nucleotides, have recently been identified as critical molecules for monitoring cancer progression. This study examined the novel lncRNAs involved in the regulation of tumor progression in breast cancer. This study identified 73 metastasis-related lncRNA candidates from comparison of paired isogenic high and low human metastatic breast cancer cell lines, and their expression levels were verified in clinical tumor samples by using The Cancer Genome Atlas. Among the cell lines, a novel lncRNA, *LOC550643*, was highly expressed in breast cancer cells. Furthermore, the high expression of *LOC550643* was significantly correlated with the poor prognosis of breast cancer patients, especially those with triple-negative breast cancer. Knockdown of *LOC550643* inhibited cell proliferation of breast cancer cells by blocking cell cycle progression at S phase. *LOC550643* promoted important *in vitro* metastatic traits such as cell migration and invasion. Furthermore, *LOC550643* could inhibit miR-125b-2-3p expression to promote breast cancer cell growth and invasiveness. In addition, by using a xenograft mouse model, we demonstrated that depletion of *LOC550643* suppressed the lung metastatic potential of breast cancer cells. Overall, our study shows that *LOC550643* plays an important role in breast cancer cell metastasis and growth, and *LOC550643* could be a potential diagnosis biomarker and therapeutic target for breast cancer.

## Introduction

Breast cancer is the most common cancer and the second leading cause of cancer deaths among women worldwide (Bray et al., 2018). Unlike other malignancies, breast cancer is considered a heterogenous disease consisting of at least four molecular subtypes, namely luminal A, luminal B, HER2, and triple negative (or basal-like) subtypes, which are determined on the basis of hormone receptor status and HER2 expression (Yeo et al., 2014). Among breast cancer subtypes, triple negative breast cancer (TNBC) is the most aggressive one, it has the poorest 5-year survival rate, and has the shortest recurrence intervals because of the lack of effective treatments and targeted therapy (Bertucci et al., 2012). Therefore, developing useful prognostic biomarkers and alternative therapeutic approaches for patients with TNBC is urgently required.

Long non-coding RNAs (lncRNAs) are an emerging class of ncRNAs with an estimated number of 15,000 human genome transcripts, and these RNAs have been demonstrated to play a crucial role in tumor development (Derrien et al., 2011; Serviss et al., 2014). Abundant evidence indicates that lncRNAs exert their function by interacting with cellular macromolecules such as chromatin, RNAs and proteins to regulate genes important for cell proliferation, motility, invasiveness, and angiogenesis (Serviss et al., 2014; Kumar and Goyal, 2017; Zhang et al., 2020). lncRNAs can also act as decoys to titrate cancer-related miRNAs to regulate the aforementioned cellular activities (Yoon et al., 2014; Chan and Tay, 2018). Despite numerous efforts being made to identify the role of lncRNA in tumor progression, the function of most lncRNAs remains largely unknown.

In this study, we compared the lncRNA expression profiles between the highly metastatic MDA-MB-231-IV2-1 subline and its parental line MDA-MB-231 to identify clinically relevant metastasis-related lncRNAs that can be found in The Cancer Genome Atlas (TCGA) database. We demonstrated that *LOC550643* promoted *in vitro* metastasis-traits such as cell growth, migration, and invasion through inhibiting miR-125b-2-3p. High *LOC550643* expression was significantly correlated with poor overall survival (OS) when compared with low *LOC550643* expression in TNBC patients.

## Materials and Methods

### Cell Line

Eight human cell lines, namely MCF-10A, MCF-7, T-47D, SK-BR-3, BT-549, Hs578T, MDA-MB-231, and MDA-MB-453, were originally obtained from the American Type Culture Collection and maintained in Dulbecco’s modified Eagle medium (DMEM) supplemented with 10% inactivated fetal bovine serum (FBS) (Invitrogen, Carlsbad, CA, United States). The highly metastatic MDA-MB-231-IV2-1 and MDA-MB-231-IV2-2 cells were isolated using an *in vivo* mouse model, as in our previous study (Chan et al., 2014). Breast cancer cell total RNA was prepared using TRIZOL (Invitrogen, Carlsbad, CA, United States) in accordance with the manufacturer’s instructions. Total RNA was incubated with DNase I (20 mg/ml) at 37°C for 30 min followed by phenol-chloroform extraction. RNA was then precipitated with isopropanol at 4°C for 30 min followed by centrifugation at 12,500 rpm. RNA pellets were washed with 70% ethanol three times and dissolved in DEPC-treated water.

### Microarray Analysis

MDA-MB-231-P and MDA-MB-231-IV2-1 cells were cultured in DMEM supplemented with 10% inactivated FBS. The cells were subjected to RNA extraction after 70% confluence was reached. Breast cancer cell total RNA was prepared using TRIZOL approach. Next, 0.2 μg of total RNA was subjected to amplification by using a Low Input Quick-Amp Labeling kit (Agilent Technologies, United States) and labeled with Cy3 (CyDye, Agilent Technologies, United States). Finally, 0.6 μg of Cy3-labled cRNA was fragmented and hybridized using an Agilent SurePrint G3 Human V2 GE 8 × 60K microarray (Agilent Technologies, United States) at 65°C for 17 h. After hybridization, the microarray image was scanned and analyzed using Feature Extraction 10.5.1.1 (Agilent Technologies, United States). We performed microarray experiments by using the Agilent oligonucleotide ChIP-on-chip protocol, then data analysis were analyzing by Welgene Biotech (Taipei, Taiwan) We submitted all microarray raw data to the National Center for Biotechnology Information (NCBI) Gene Expression Omnibus (GEO), and they are freely available (accession number: GSE175513).

### Pathway Enrichment Analysis

The differentially expressed gene–associated networks were analyzed with *Kyoto Encyclopedia of Genes and Genomes* (KEGG) pathways. Altered gene expressions were selected from microarray data and were subsequently fed into the KEGG pathways using the R package SubPathwayMiner (version 3.1). Enriched pathways were extracted by Hypergeometric testing the false discovery rate–corrected *q*-value were calculated.

### Reverse Transcription and Real-Time Polymerase Chain Reaction (PCR) Analysis

Total RNA was reverse-transcribed with random primers and SuperScript III Reverse Transcriptase in accordance with the relevant user manual (Invitrogen, Carlsbad, CA, United States). The RT reaction was carried out at 42°C for 1 h followed by inactivation at 70°C for 10 min. The cDNA was used for the subsequent PCR reaction with gene-specific primers, and gene expression analysis was conducted using a SYBR Green I assay (Applied Biosystems, Foster City, CA, United States). Delta-delta Ct values were used to determine their relative expressions as fold changes using GAPDH as an internal control. The primers used are listed in Supplementary Table 1.

### Clinical Samples

The data and specimens used in this study were collected from 36 breast cancer patients who underwent surgery at the Department of Surgery, Kaohsiung Veterans General Hospital (KSVGH), Taiwan. Informed consent forms were obtained from all patients by the KSVGH biobank. This study was approved by the ethics committees of KSVGH (VGHKS16-CT10-08).

### *LOC550643* Expression Analysis and Clinical Impact of TCGA Database

In this study, we downloaded TCGA data on RNA sequences in breast cancer tissue samples from the TCGA online database^1^. The RNA-seq data of 1092 breast cancer tissue samples and 113 corresponding adjacent normal tissue samples were fetched from TCGA public domain. Among them are expression profiles of 56 paired normal/tumor tissues from the breast cancer patients. We identified lncRNAs differentially expressed in cancer tissues versus their adjacent normal tissues from the 56 breast cancer patients. The clinical information of the patients was also downloaded. In this study, the Kaplan–Meier survival analysis was applied for evaluating *LOC550643* relevant overall survival in 1070 breast cancer patients.

### RNA Ligase–Mediated Rapid Amplification of the 5′ and 3′ Rapid Amplified cDNA Ends for Full-Length Determination

To map the full *LOC550643* sequence, RNA ligase–mediated rapid amplification of cDNA ends (RACE) was performed using the GeneRacer kit (Invitrogen, Carlsbad, CA, United States) in accordance with the manufacturer’s instructions. The 5′ RACE was conducted to obtain the complete sequence of the lncRNA transcript. The 5′ end sequence of the lncRNA transcript was generated using GeneRacer 5′ primer and reverse primers. In addition, the 3′ end sequence of the lncRNA transcript was obtained using the GeneRacer 3′ primer and forward primers. The complete sequence of the lncRNA transcript was obtained by merging the 5′ end and 3′ end sequences. PCR products were gel-purified and cloned into a pCR4 TOPO vector (Invitrogen, Carlsbad, CA, United States) for sequencing. The primers used in the RNA ligase–mediated RACE are presented in Supplementary Table 1.

### *LOC550643* Knockdown

MDA-MB-231-IV2-1 cells with approximately 70% confluence were cultured in 60-mm cell culture dishes with DMEM supplemented with 10% FBS for 24 h prior to transient transfection. For *LOC550643* knockdown, *LOC550643* siRNAs (10 nmol/L) transfection was conducted using lipofectamine RNAiMAX (Invitrogen) in Opti-MEM (Invitrogen). The sequences of siRNAs purchased from Gene Discovery are presented in Supplementary Table 2.

### Cell Proliferation Assay

Cells were transfected with either si-*LOC550643* or siNC and plated onto 96-well plates. Cell proliferation was determined at the time point of 0, 1, 2, 3, and 4 days. Cell viability was analyzed by MTS assay (Promega Corporation, United States) in accordance with the manufacturer’s instructions.

### Colony Formation Assay

A total of 4000 cells were plated in 6-well plates and incubated at 37°C for 10 days. The cultured medium was replaced every 3 days. Cells were fixed with 4% formaldehyde for 2 min, and colonies were stained with 0.5% crystal violet solution for 2 h. The 6-well plates were washed with H_2_O and air-dried. The crystal violet-stained cells were then lysed with 1 mL of 10% acetic acid followed by analysis of 595 nm the absorbance using a spectrophotometer.

### Cell Synchronization

MDA-MB-231-IV2-1 cells were synchronized at the late G1/early S phase by using a double thymidine block in accordance with a previous study (Chen and Deng, 2018). MDA-MB-231-IV2-1 cells with approximately 70% confluence were seeded in 6-cm dishes and transfected with si-*LOC550643* as previously described. After 24 h of siRNA transfection, the first thymidine block was initiated through the addition of thymidine (Sigma) to the wells at a final concentration of 2 mM for 16 h. Cells were then washed with 1X phosphate-buffered saline (PBS) and further cultured in normal cell maintenance media for 8 h to release the cell cycle. The second 16-h thymidine (2 mM) block was initiated immediately following the 8-h cell cycle release. After the second thymidine block, the cells were washed with 1X PBS to release the cell cycle and further incubated in a medium containing nocodazole (1 μg/mL) to arrest cells in the G2/M phase. The cell cycle progression of the cells was analyzed using flow cytometry every hour for 8 h following the release. The cell cycle profiles were analyzed using NucleoView NC-3000 to determine the percentages of cells in the G1, S, and G2/M phases.

### Image Flow Cytometry Assay

The cell cycle of MDA-MB-231-IV2-1 with *LOC550643* knockdown was determined using the fluorescence image cytometer NucleoCounter NC-3000 (ChemoMetec, Gydevang, Lillerød, Denmark). After transfection with siRNA for 48 h, the cells were trypsinized and the number of cells was determined. A total of 1 × 10^6^ cells were fixed with 70% ethanol at 4°C overnight. The cells were then stained with 1 μg/mL DAPI (4’,6-diamidino-2-phenylindole) solution containing DAPI and 0.1% triton X-100 in PBS at 37°C for 5 min. Finally, the stained cells were analyzed using the NucleoView NC-300 software program (ChemoMetec, Gydevang, Lillerød, Denmark).

### Western Blotting

The cells were harvested 48 h after transient transfection followed by PBS wash, and treated with lysis buffer (50 mM Tris-HCl at pH 8.0, 150 mM NaCl, 1% NP-40, 0.02% sodium azide, 1 μg/mL aprotinin, 1 mM PMSF) at 4°C for 30 min. The relevant details are described in our previous study (Tsai et al., 2018). Detailed information regarding the primary antibodies used in this study is presented in Supplementary Table 3.

### Cell Invasion

For the invasion assay, 3 × 10^5^ cells were suspended in 200 μL of DMEM with 2% FBS and plated onto BD BioCoat Matrigel Invasion Chambers (24-well insert; pore size, 8 μm; BD Biosciences) precoated with 100 μL Matrigel (0.5 μg/μL) per insert and incubated in a humidified chamber for 2 h at 37°C. The lower chamber was filled with DMEM containing 10% FBS. After being cultured for 24 h, the transwell inserts were fixed and stained with crystal violet solution (0.5% crystal violet, 5% formaldehyde, 50% ethanol, and 0.85% sodium chloride). Cells that did not invade were erased with a cotton swab. The invaded cells were imaged under a microscope at 100 × magnification.

### Animal Model

A xenograft mouse model was performed in this study, 10 mice were used for the lung metastasis assay (five controls and five for LOC550643 knockdown). A total of 1 × 10^6^ cells were transfected with the indicated siRNAs 24 h before injection, and then they were suspended in 100 μL of 1X PBS and were injected into C.B-17 severe combined immunodeficient (SCID) mice via tail vein. The lung metastasis status of the mice was investigated 3 weeks after injection. The mice were intravenously injected with 200 μL of luciferin (20 mg/mL) and anesthetized with isoflurane. The mice were then transferred to the imaging chamber of the *in vivo* imaging system (IVIS) spectrum to obtain the luminescent signals (Perkin Elmer Inc., MA, United States). The luminescent signals were measured and analyzed using Living Image 4.4 (Perkin Elmer Inc., MA, United States).

### *In vivo* Imaging System

The mice were injected intravenously with 200 μL of luciferin (20 mg/mL) at a dose of 200 mg/kg and then euthanized using CO_2_. The mouse lungs were surgically removed and placed in the IVIS chamber for IVIS imaging between 5 and 15 min after sacrifice. The bioluminescence images were acquired using the IVIS SpectrumCT and were analyzed using Living Image 4.4 (Perkin Elmer Inc., CA, United States).

### Hematoxylin and Eosin Staining

Samples were fixed in 4% paraformaldehyde for 1 h at room temperature and then embedded in paraffin. The 6-μm tissue sections were prepared using a microtome followed by deparaffinization with xylene. The tissue sections were slowly rehydrated by immersing them into decreasing concentrations of ethanol and then placing them in deionized water for the subsequent hematoxylin and eosin (H&E) staining. The tissue sections were then stained with hematoxylin solution (Merck, CA, United States) for 3 min followed by washing with water for 5 min. Finally, the tissue sections were stained with eosin (Merck, CA, United States) for 30 s followed by dehydration using increasing concentrations of ethanol. The sections were finally maintained in xylene. The tissue sections were mounted using Micromount (Leica, CA, United States) for 1 h at room temperature.

### Small RNA Transcriptome Analysis Through Next-Generation Sequencing

After the MDA-MB-231-IV2-1 cells were transfected with si-*LOC550643* and the scrambled control for 48 h, the total RNA was extracted from two samples by using TRIZOL reagent. The small RNA library was prepared using the NEBNext small RNA library prep kit (New England Biolabs). The library preparation process is described in details in our previous study (Tseng et al., 2017). Finally, the small RNA profiles of the MDA-MB-231-IV2-control and MDA-MB-231-IV2-LOC550643 knockdown were performed using the MiSeq V2 reagent kit (150 cycles; Illumina, San Diego, CA, United States). The sequencing data were analyzed using our own tool (Pan et al., 2014). All microarray raw data were deposited in the NCBI GEO, and they are all accessible (accession number: GSE175514).

### MicroRNA Expression Analysis and Clinical Impacts of TCGA Database

We downloaded small RNA expression data for breast cancer tissues from TCGA database. The expression profiles of 778 breast cancer tissue samples and 87 matched normal parts were fetched from TCGA portal. The clinical information of breast cancer patients was also downloaded. Small RNA and RNA sequencing were performed simultaneously for 757 breast cancer patients. The small RNA transcriptome profiles were subject to an OS analysis by using the Kaplan–Meier method.

### Stem-Loop Reverse Transcription PCR

The PCR process is described in detail in our previous study (Liu et al., 2018). The miR-125b-2-3p expression levels were normalized to those of U6 small RNA (ΔCt = target miR-125b-2-3p Ct-U6 Ct). The primers used are presented in Supplementary Table 1.

### Ectopic Expression of miRNAs

Breast cancer cells were transfected with 10 nM miRNA-125b-2-3p mimics or a scrambled control (GenDiscovery Biotechnology Inc., Taiwan) by using lipofectamine RNAiMAX reagent. After 24 h of transfection, the miR-125b-2-3p expression levels were confirmed using stem-loop reverse transcription quantitative PCR.

### Candidate miRNA Targets and Luciferase Activity Assay

The putative miRNAs targeting LOC550643 (Genebanl ID: MH892397) were predicted using TargetScan. In this study, 189 miRNAs were predicted to bind to LOC550643. The full lengths of LOC550643-wt (Genebanl ID: MH892397) and LOC550643-mut (mutated miR-125b-2-3p binding site) were synthesized using Invitrogen (Invitrogen, Waltham, MA, United States). The fragments were cloned into the pMIR-REPORT vector. During synthesis, a *Spe*I cutting site was added at the 5′ end, and *Hin*dIII was added at the 3′ end of the LOC550643-wt and LOC550643-mut fragments, which were then cloned into the pMIR-REPORT vector. Subsequently, the pMIR–LOC550643-wt or pMIR–LOC550643-mut vectors were cotransfected separately with miR-125b-2-3p mimics or scrambled controls into breast cancer cells by using lipofectamine RNAiMAX reagent. 24 h post transfection, cells were harvested with lysis buffer and luciferase activity was measured by using the Dual-Glo Luciferase Reporter Assay System (Promega Corporation, Madison, WI, United States).

### Statistical Analysis

The clinical impacts of *LOC550643* on breast cancer were evaluated using Fisher’s test and a chi-squared test. *LOC550643* expression levels were tested in triplicate for clinical samples, cell growth, invasion ability, colony formation, cell cycle, and reporter experiments. The histograms present the mean values, and the error bars indicate the standard deviation. The *in vitro* and animal experiment data were analyzed using Student *t*-tests. OS was determine using the log-rank test or the Kaplan–Meier method. A *p*-value of < 0.05 was considered significant for the experiments.

## Results

### Identification of Metastasis-Related lncRNA Candidates in TNBC

**FIGURE 1:**
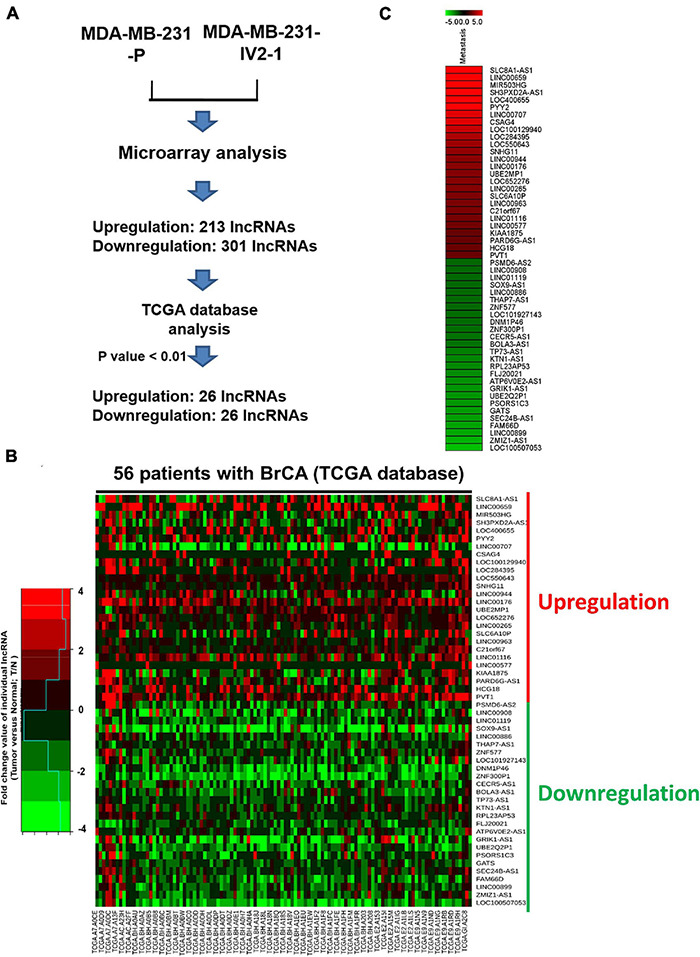
Comparison of MDA-MB-231-P and MDA-MB-231-IV2-1 lncRNA profiles. **(A)** Flowchart for the microarray approach to screening lncRNA candidates and the use of TCGA database to examine their expression levels. **(B)** The 56 expression profiles of normal/tumor tissue pairs from patients with breast cancer obtained from TCGA database. The fold change values of the individual lncRNAs were calculated using the lncRNA expression levels of tumor cells compared with non-tumor cells. A heatmap of the differential expression of the 52 lncRNA candidates in 56 patients with breast cancer is presented. **(C)** Heatmap of selected putative metastasis-associated lncRNAs in MDA-MB-231-P cells compared with MDA-MB-IV2-1 cells.

**FIGURE 2:**
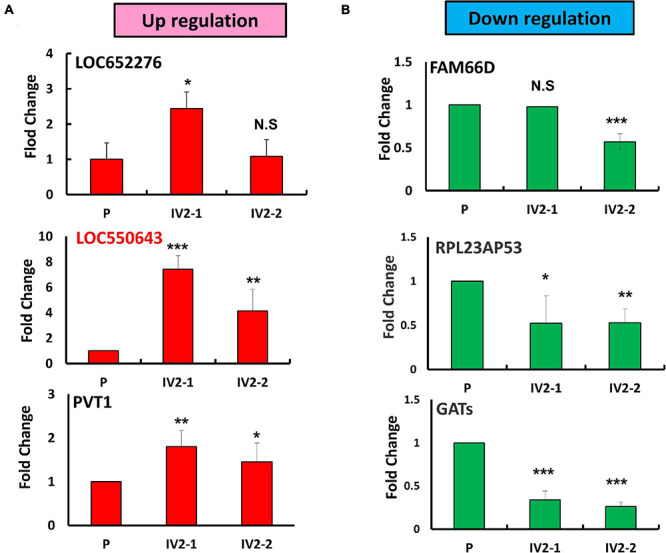
Identification of metastasis-related lncRNA candidates in MDA-MB-231-IV2 cells. **(A)** Expression levels of the three putative oncogenic lncRNA candidates examined in the parental MDA-MB-231, MDA-MB-231-IV2-1, and MDA-MB-231-IV2-2 cells. **(B)** Expression levels of the three tumor-suppressive lncRNA candidates examined in the parental MDA-MB-231-P, MDA-MB-231-IV2-1, and MDA-MB-231-IV2-2 cells. All experiments were carried out in triplicate. Using Student’s *t*-test to analyze our data and *p* < 0.05 was considered significant (*p < 0.05, **p < 0.01, and ***p < 0.001).

To explore the role of lncRNAs in breast cancer metastasis, we first generated expression profiles of human lncRNAs in MDA-MB-231-P and MDA-MB-231-IV2-1 cells by using a microarray approach (Agilent SurePrint G3 Human V2 GE; 34092 protein-coding genes and 8715 lncRNAs). The highly metastatic MDA-MB-231-IV2-1 cells were isolated using an *in vivo* mouse model; therefore, the MDA-MB-231-IV2-1 cells exhibited higher metastatic ability than did the MDA-MB-231-P cells (Chan et al., 2014). After the microarray profiling process, several protein-coding genes with differential expression were identified between the MDA-MB-231-P and MDA-MB-231-IV2-1 cells (2926 upregulated genes and 2989 downregulated genes; fold change > 2 and < 0.5, *p* < 0.01; Supplementary Figures 1A,B). These raw microarray data were uploaded to the NCBI GEO database and are freely available (accession number: GSE175513). Through pathway enrichment analysis, we observed that these differentially expressed genes were significantly involved in cell migration and motility signaling pathways (Supplementary Figure 1C). We also identified several differentially expressed lncRNA candidates between the MDA-MB-231-P and IV2-1 cells (213 upregulated lncRNAs and 301 downregulated lncRNAs; fold change: >2 and <0.5, respectively, *p* < 0.01; Figure 1A). We further examined the expression levels of these lncRNA candidates (514 differentially expressed lncRNAs) in breast cancer cells by using TCGA database. A total of 52 lncRNA candidates had significant differential expression in breast cancer cells when compared with adjacent normal cells (Figure 1B). Among them, we first randomly selected 6 lncRNA candidates to confirm their expression levels by using real-time PCR with MDA-MB-231-P, MDA-MB-231-IV2-1, and MDA-MB-231-IV2-2 cells. Similar to the MDA-MB-231-IV2-1 cells, the MDA-MB-231-IV2-2 cells were derived from MDA-MB-231-P cells by using an *in vivo* mouse model (Chan et al., 2014). Our data revealed that the expression levels of LOC550643 and PVT1 were significantly upregulated in MDA-MB-231-IV2-1 and MDA-MB-231-IV2-2 cells (Figure 2A), whereas the expression levels of *LOC652276* were significantly increased in MDA-MB-231-IV2-1 cells but not in MDA-MB-231-IV2-2 cells when compared with MDA-MB-231-P cells. In addition, the expression levels of *PRL23AP53* and *GATS* were significantly lower in the MD-MB-231-IV2-1 and MDA-MB-231-IV2-2 cells, but those of *FAM66D* were significantly lower only in MD-MB-231-IV2-2 cells (Figure 2B).

### Correlation of *LOC550643* Expression With Poor Prognosis in Breast Cancer Patients

**FIGURE 3:**
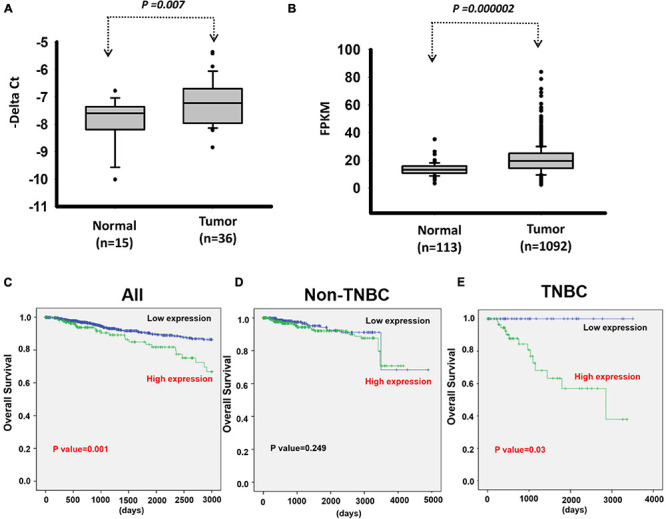
Analysis of *LOC550643* expression levels in breast cancer tissues. **(A)** Expression levels of *LOC550643* in breast cancer tissues and corresponding adjacent normal tissues (real-time PCR). **(B)** Expression levels of *LOC550643* in human breast cancer tissues versus normal tissues (TCGA database). **(C–E)** Impacts of *LOC550643* expression on the OS of TNBC patients versus non-TNBC patients.

We next investigated the clinical impacts of the 52 metastasis-associated lncRNAs by analyzing TCGA data. Among them, a novel lncRNA, *LOC550643*, was correlated with a survival curve in patients with breast cancer (Supplementary Figure 2). As presented in Figures 3A,B, the expression levels of *LOC550643* were significantly upregulated in breast cancer tissue when compared with adjacent normal tissues. Moreover, high LOC550643 expression levels were associated with pathological stages (*p* = 0.009) and weakly associated with pN stage (*p* = 0.053) (Table 1). The Kaplan–Meier analysis revealed that high *LOC550643* expression levels were significantly correlated with a poor OS curve (effect of *LOC550643* on OS: *p* = 0.003, Table 2 and Figure 3C). A multivariate Cox regression model indicated a significant association between high *LOC550643* expression levels and poor OS (*LOC550643*: adjusted hazard ratio, 2.00; 95% CI, 1.24–3.23; *p* = 0.004; Table 2). Stratified by molecular subtypes, the Kaplan–Meier survival analysis indicated that when compared with non-TNBC patients, high *LOC550643* expression was significantly associated with poor OS in patients with TNBC (Figures 3D,E). Therefore, we surmised that *LOC550643* might participate in the cellular machinery responsible for regulating growth and metastasis of breast cancer.

**TABLE 1:** The relationship between expression levels of *LOC550643* and clinicopathologic data of BC patients.

	**Expression level of *LOC550643* (*n* = 1073)**		
**Variables**	**Low expression**	**Highly expression**	***P*-value**
		
	**Number (%)**	**Number (%)**	
AJCC pathological stage			
I	243 (89.0)	30 (11.0)	0.009^*b*^
II	451 (81.3)	104 (18.7)	
III	191 (84.9)	34 (15.1)	
IV	14 (70.0)	6 (30.0)	
pT stage			
T1	308 (86.5)	48 (13.5)	0.267^*b*^
T2	463 (82.5)	98 (17.5)	
T3	105 (83.3)	21 (16.7)	
T4	23 (76.7)	7 (23.3)	
pN stage (*n* = 1067)			
N0	494 (85.3)	85 (14.7)	0.053^*a*^
N1	246 (79.4)	64 (20.6)	
N2	92 (85.2)	16 (14.8)	
N3	63 (90.0)	7 (10.0)	
pM stage			
M0	885 (84.0)	168 (16.0)	0.118^*b*^
M1	14 (70.0)	6 (30.0)	

*^a^p-value is estimated by chi-square test.*

*^b^p-value is estimated by Fisher’s test.*

**TABLE 2:** Univariate and multivariate Cox’s regression analysis of *LOC550643* and expression for overall survival of 1070 patients with breast cancer.

		**OS**		
**Characteristic**	**No. (%)**	**CHR (95% CI)**	***P*-value**	**AHR (95% CI)**	***P*-value**
*LOC550643*	(*n*=)				
Low	896 (83.7)	1.00		1.00	
High	174 (16.3)	2.15 (1.34–3.46)	0.003	2.00 (1.24–3.23)	0.004

*Abbreviation: OS, overall survival; CHR, crude hazard ratio; AHR, adjusted hazard ratio.*

*AHR were adjusted for AJCC pathological stage (II, III, and IV VS. I).*

### Identification of the Full Length *LOC550643* in Breast Cancer Cells

According to the University of Santa Cruz (UCSC) database, LOC550643 comprises three exons (Supplementary Figure 3A). However, the real length of the LOC550643 sequence is unclear; therefore, we identified their full lengths by performing 5’ and 3’ RACE. The RACE results revealed three *LOC550643* transcripts of varying lengths (V1: 718 bp; V2: 581 bp; and V3: 476 bp) (Supplementary Figure 3B and Supplementary Table 4). Details on the gene structures of the three *LOC550643* isoforms are presented in Supplementary Figures 3C,D. Despite the aforementioned discovery, the detailed roles of *LOC550643* and its isoforms in breast cancer metastasis are unclear. We also examined *LOC550643* expression among breast cancer cell lines with different molecular subtypes and invasive capabilities (Supplementary Figure 4A). To address the function of *LOC550643* in breast cancer cells, we designed two siRNAs targeting *LOC550643* with sequences complementary to the second and third exons, respectively (Supplementary Figure 4B). After siRNA transfection, the knockdown efficiency of *LOC550643* in the MDA-MB-231-IV2-1 cells was confirmed using real-time PCR. The endogenous expression levels of *LOC550643* in scramble siRNA-transfected cells (N.C) were similar to that of in the mock control group (Supplementary Figure 4C). However, the *LOC550643* mRNA levels in the MDA-MB-231-IV2-1 cells markedly decreased after si-*LOC550643*#301 or si-*LOC550643*#543 transfection (Supplementary Figure 4C).

### Suppression of Breast Cancer Cell Growth Through the Impairment of Cell Cycle Progression by *LOC550643* Knockdown

**FIGURE 4:**
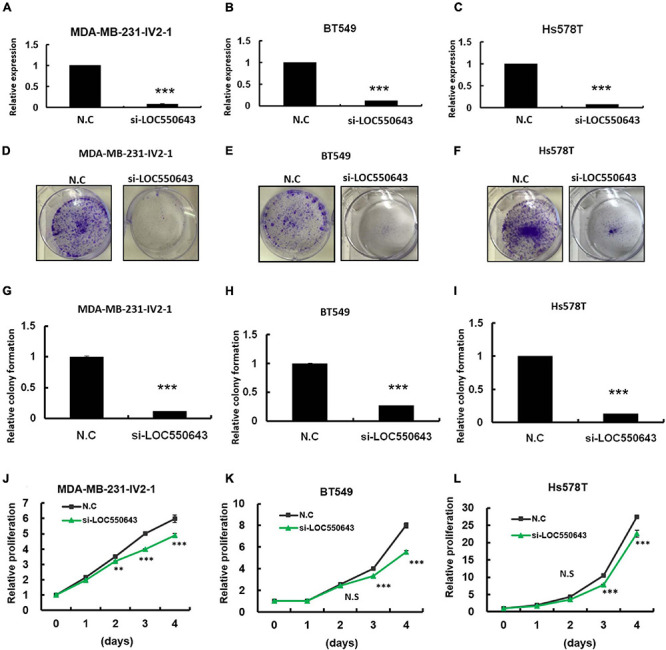
Examination of *LOC550643* cellular function in breast cancer cell lines. The siRNAs (N.C, si-*LOC550643*-pool) were individually transfected into breast cancer cells (MDA-MB-231-IV2-1, BT549, and Hs578T) followed by cellular function examination. **(A–C)** Relative expression of *LOC550643* in three breast cancer cell lines after siRNA transfection (real-time PCR). **(D–F)** Colony formation assay performed for MDA-MB-231-IV2-1, BT549, and Hs578T cells after transfection with si-*LOC550643* or a scramble control. The cells were fixed and stained with crystal violet solution. **(G–I)** Relative colony formation ability quantified using a 595-nm optical density. **(J–L)** Cell proliferation measured using the CellTiter-Glo assay at various time points following *LOC5506431* knockdown (0, 1, 3, and 4 days) as compared with the scramble control. All experiments were carried out in triplicate. Using Student’s *t*-test to analyze our data and *p* < 0.05 was considered significant (*p < 0.05, **p < 0.01, and ***p < 0.001).

**FIGURE 5:**
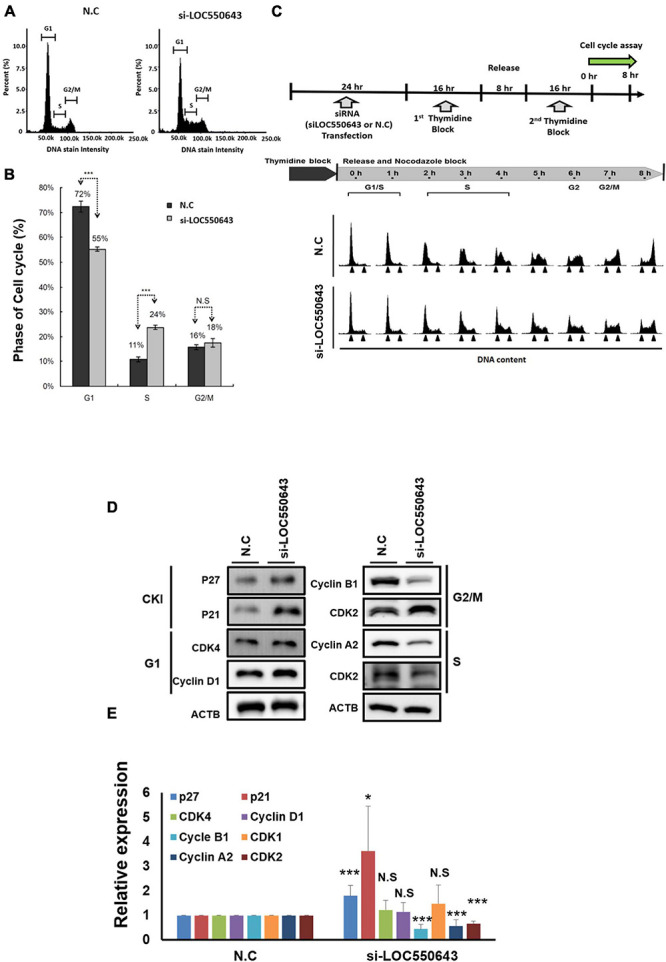
Suppression of breast cancer cell growth through the induction of cell cycle arrest at the S phase following *LOC550643* depletion. **(A)** Cell cycle of MDA-MB-231-IV2-1 cells after 48 h of *LOC550643* knockdown. **(B)** Experiments were performed in triplicate and data were quantified and analyzed using Student’s *t*-test. **(C)** Cell cycle of MDA-MB-231-IV2-1 cells synchronized using a double thymidine block and released into nocodazole medium for the indicated times. At each time point, the cells were subjected to cell cycle analysis. **(D)** Expression levels of cell cycle–related genes examined using Western blotting assays following 48 h of siRNA transfection. **(E)** Western blotting assays were done in triplicate, then all experiment data were further quantified. Using Student’s *t*-test to analyze our data and *p* < 0.05 was considered significant (*p < 0.05, **p < 0.01, and ***p < 0.001).

As shown above, high *LOC550643* expression was correlated with poor OS in breast cancer patients, particularly in those with TNBC (Figure 3E). Furthermore, we pooled two siRNAs (siRNA#301 and siRNA#543), which could reduce the overall concentration and prevent off-target effects for examining the biological function of *LOC550643* in TNBC cell lines. After transfection with the pooled siRNAs, *LOC550643* expression levels were obviously reduced in MDA-MB-231-IV2-1, BT549, and Hs578T cells (Figures 4A–C). Our data indicated that *LOC550643* knockdown significantly suppressed colony formation capability in the three breast cancer cell lines (Figures 4D–I). Furthermore, *LOC550643* knockdown also slightly inhibited the proliferation of breast cancer cells (Figures 4J–L). These results implied that *LOC550643* might be involved in breast cancer cell growth. To more thoroughly understand the detail mechanism of the involvement of *LOC550643* in breast cancer cell growth, we investigated the effects of *LOC550643* on cell cycle by using an image flow cytometry assay. Our data revealed a significantly increase in the S phase (13% increase, *p* < 0.001) accompanied by a 17% decrease (*p* < 0.001) of MDA-MB-231-IV2-1 G1-phase cells upon *LOC550643* knockdown (Figures 5A,B). We further checked cell cycle progression over time following release from a double thymidine block. As shown in Figure 5C, most control cells entered the G2/M phase from the late G1/early S phase 8 h after release, whereas the *LOC550643* knockdown cells were blocked in the S phase and delayed entry into the G2/M phase. We also examined the cell cycle–related genes and observed that *LOC550643* knockdown resulted in lower Cyclin B1, Cyclin A2, and CDK2 protein levels in MDA-MB-231-IV2-1 cells and increased P21 and P27 protein levels in MDA-MB-231-IV2-1 cells (Figures 5D,E). These data indicate that *LOC550643* knockdown could suppress cell growth by promoting cell cycle S phase arrest.

### Suppression of Breast Cancer Cell Lung Metastasis Potential Through *LOC550643* Depletion

**FIGURE 6:**
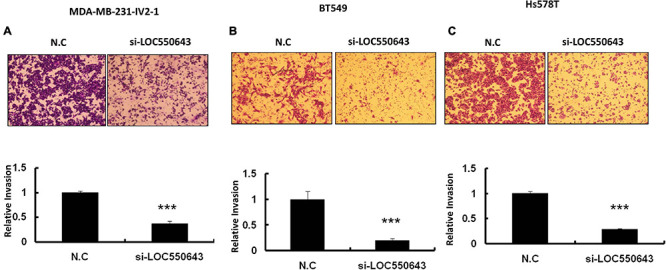
Significant suppression of breast cancer cell invasion ability through *LOC550643* knockdown. **(A–C)** Invasion assay conducted with MDA-MB-231-IV2-1, BT549, and Hs578T with and without *LOC550643* knockdown. The representative images of invading cells stained with crystal violet solution (upper panels). Three different fields were subjected to count numbers of invading cells. The related invasion capability was evaluated using the number of invading cells of *LOC550643* knockdown as compared with the control (means ± SD) (lower panels). All experiments were carried out in triplicate. Using Student’s *t*-test to analyze our data and *p* < 0.05 was considered significant (*p < 0.05, **p < 0.01, and ***p < 0.001).

**FIGURE 7:**
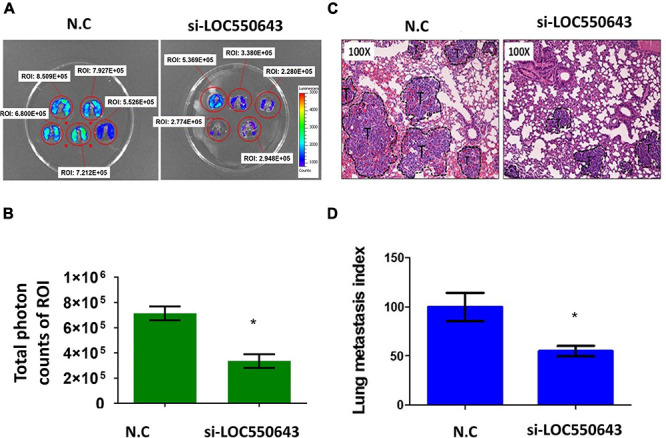
Suppression of the lung metastasis potential of MDA-MB-231-IV2-1 cells through *LOC550643* depletion. **(A)** Lung metastatic ability of si-*LOC550643* and N.C control MDA-MB-231-IV2-1 cells examined using the SCID mice model through intravenous injection. Ten SCID mice were used (five as controls and five with *LOC550643* knockdown). Three weeks after injection, lung metastasis status was assessed using IVIS. **(B)** ROI intensity calculated and analyzed using Student’s *t*-test. **(C)** Metastatic lungs examined using H&E staining. **(D)** Lung metastasis index assessed using Student’s *t*-test. *p < 0.05.

Furthermore, *LOC550643* knockdown also suppressed breast cancer cell invasion ability in three TNBC cell lines, namely MDA-MB-231-IV2-1 (Figure 6A), BT549 (Figure 6B), and Hs578T (Figure 6C). To validate our *in vitro* findings, we employed a mouse model to determine whether *LOC550643* would affect the lung metastasis potential of the MDA-MB-231-IV2-1 cells. One million *LOC550643*-depleted MDA-MB-231-IV2-1 cells labeled with luciferase and the control MDA-MB-231-IV2-1 cells labeled with luciferase were, separately, intravenously injected into SCID mice. Three weeks after injection, the mice were sacrificed, and lung metastasis were examined using IVIS imaging. The lungs of mice injected with *LOC550643*-depleted MDA-MB-231-IV2-1 cells displayed reduced luminescence signals when compared with the control group (Figures 7A,B). After IVIS imaging, the mouse lungs were fixed with 3.5% formaldehyde and embedded with paraffin to form tissue blocks. H&E staining of lung sections confirmed that the *LOC550643*-depleted MDA-MB-231-IV2-1 cells formed smaller metastases than did the control cells (Figures 7C,D). Taken together, the results revealed that *LOC550643* knockdown could suppress breast cancer cell metastasis.

### Direct Interaction of *LOC550643* With miR-125b-2-3p to Suppress Breast Cancer Cell Growth and Motility

**FIGURE 8:**
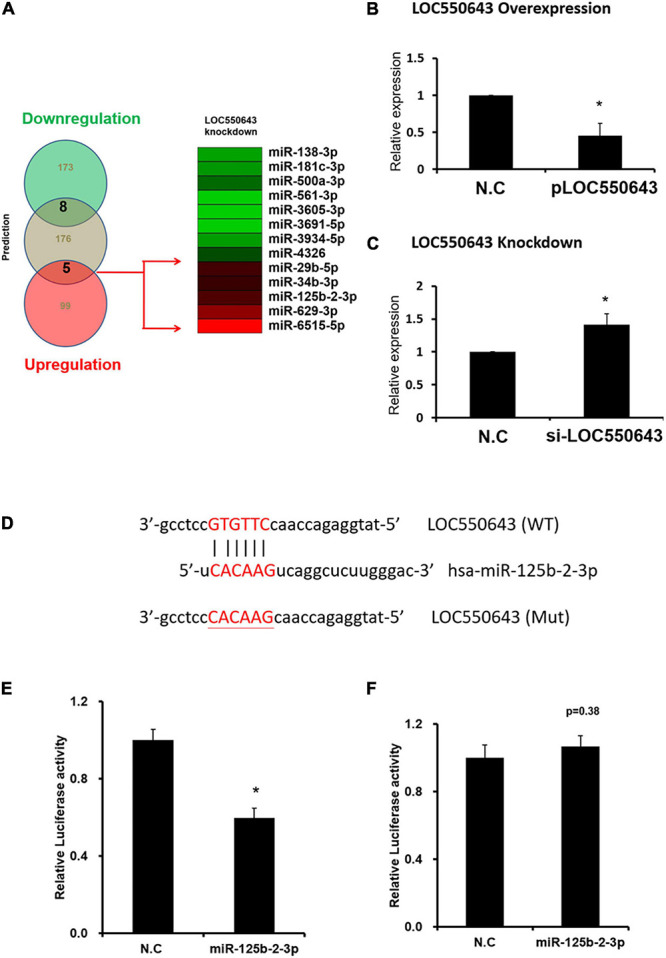
Regulation of miR-125b-2-3p expression through direct interaction with *LOC550643*. **(A)** Identification of *LOC550643*-sponged miRNA candidates through TargetScan and small RNA profiles. **(B)** Expression levels of miR-125b-2-3p examined in MDA-MB-231-P cells with ectopic *LOC550643* expression (real-time PCR). **(C)** Expression levels of miR-125b-2-3p assessed in MDA-MB-231-IV2-1 cells with si-*LOC550643* transfection by real-time PCR. **(D)** Schema of luciferase constructs. The miR-125b-2-3p targeting sequence in the *LOC550643* sequence is represented in the upper panel, and the mutated sequence of its binding region is in red. **(E,F)** Relative luciferase activity of pMIR-LOC550643-wt and pMIR-LOC550643-mut identified in breast cancer cells transfected with miR-125b-2-3p mimics and scramble control. All experiments were carried out in triplicate. Using Student’s *t*-test to analyze our data and *p* < 0.05 was considered significant (*p < 0.05, **p < 0.01, and ***p < 0.001).

**FIGURE 9:**
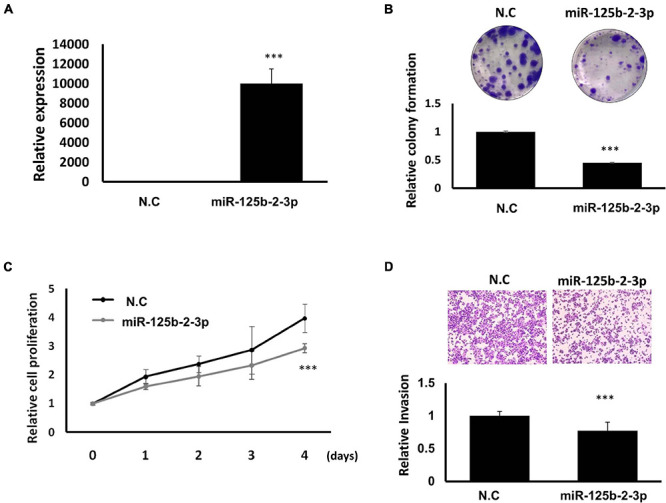
Suppression of breast cancer cell growth and motility through miR-125b-2-3p expression. **(A)** Expression levels of miR-125b-2-3p examined in MDA-MB-231-IV2-1 cells after miR-125b-2-3p mimics transfection (real-time PCR). **(B)** Colony formation ability examined and quantified in MDA-MB-231-IV2-1 cells with or without miR-125b-2-3p overexpression. **(C)** Cell proliferation of MDA-MB-231-IV2-1 cells assessed after miR-125b-2-3p transfection for various time periods. **(D)** Invasion capability assessed using the transwell assay in MDA-MB-231-IV2-1 cells transfected with miR-125b-2-3p and the scramble control. The cell images of a representative experiment are displayed. The values quantified using Ascent are in the graph. Data are reported as the numbers of invading cells relative to the controls (means ± SD). All experiments were carried out in triplicate. Using Student’s *t*-test to analyze our data and *p* < 0.05 was considered significant (*p < 0.05, **p < 0.01, and ***p < 0.001).

To further explore the mechanism of how *LOC550643* modulates breast cancer cell growth and motility, we performed miRNA transcriptome analysis using breast cancer cells with and without *LOC550643* knockdown. The transcriptome data analysis revealed 181 downregulated and 104 upregulated miRNAs in MDA-MB-231-IV2-1 cells upon *LOC550643* knockdown (Figure 8A). In addition, target prediction tools identified 189 miRNAs directly bound to *LOC550643* sequences (Figure 8A). Relevant studies have revealed that the target-directed miRNA degradation mechanism (TDMD) could modulate miRNA stability by binding target genes. Furthermore, the transcripts of target genes with highly complementary miRNA-binding sites could induce miRNA degradation (Bitetti et al., 2018; Ghini et al., 2018). In accordance with TDMD theory, we suggest that *LOC550643* knockdown could increase abundance of target miRNA candidates by reducing *LOC550643*–miRNA interactions in MDA-MB-231-IV2-1 cells. We identified 5 miRNAs, namely miR-29b-5p, miR-34b-3p, miR-125b-2-3p, miR-629-3p, and miR-6515-5p, that could be sponged by *LOC550643*. We further analyzed the expression levels of miR-29b-5p, miR-34b-3p, miR-125b-2-3p, and miR-629-3p in breast cancer cells and observed that only miR-125b-2-3p was significantly lower in breast cancer tissues compared with the normal tissues (Supplementary Figure 5). Moreover, miR-125b-2-3p expression was greatly decreased in breast cancer cells upon *LOC550643* overexpression, whereas miR-125b-2-3p expression levels were significantly higher in *LOC550643*-depleted breast cancer cells (Figures 8B,C). Therefore, we examined whether miR-125b-2-3p would directly interact with *LOC550643*. To do so, the full length *LOC550643* was cloned immediately downstream of the luciferase reporter, and the luciferase activity was measured in the presence of miR-125b-2-3p or a scramble sequence. As presented in Figure 8E, luciferase reporter assay revealed that the luciferase activity levels of 293T cells cotransfected with miR-125b-2-3p mimics and the luciferase reporter plasmid were significantly lower than those of cells cotransfected with the control miRNA mimics. The miR-125b-2-3p mimics had no inhibitory effect on the luciferase activity of the reporter plasmid containing *LOC550643* with mutated miR-125b-2-3p binding sites (Figure 8F). On the basis of these findings, we concluded that *LOC550643* is a miRNA sponge for miR-125b-2-3p in breast cancer cells. Next, we examined the role of miR-125b-2-3p in breast cancer cells and observed that miR-125b-3p expression significantly suppressed cell proliferation, colony formation, and invasion ability (Figures 9A–D). Low miR-125b-2-3p expression is associated with poor survival in patients with breast cancer (Supplementary Figure 6A). Because *LOC550643* exerts its oncogenic function through miR-125b-2-3p sponging, we explored the impact of *LOC550643*–miR-125b-2-3p axis activity in breast cancer cells. Patients were classified into three groups according to LOC550643 and miR-125b-2-3p expression. The first group comprised patients with low *LOC550643* and high miR-125b-2-3p expression, the second group comprised patients with both high or both low *LOC550643* and miR-125b-2-3p expression, and the third group comprised patients with high *LOC550643* and low miR-125b-2-3p expression. As presented in Supplementary Figure 6B, breast cancer patients with high *LOC550643* and low miR-125b-2-3p expression had the poorest survival rate among the three groups. Therefore, we conclude that *LOC550643* promotes breast cancer cell growth and metastasis through miR-125b-2-3p sponging.

## Discussion

lncRNA is no longer considered “transcription noise,” and its abnormal expression was revealed to be meaningful to the onset and development of human malignancies (Schmitt and Chang, 2016). Thus, lncRNA has become one of the major topic in cancer research. A number of studies have recently indicated that numerous lncRNAs are associated with breast cancer progression, including *GAS5* (Mourtada-Maarabouni et al., 2009; Zheng et al., 2020), *H19* (Adriaenssens et al., 1998), *HOTAIR* (Rinn et al., 2007), *MALAT1* (Loi et al., 2007), *NEAT1* (Silva et al., 2011), *UCA1* (Wang et al., 2008), *XIST* (Benoît et al., 2007), and *BCAR4* (Peng et al., 2021). In our study, we performed transcriptome analysis of MDA-MB-231-P and MDA-MB-231-IV2-1 cells and identified several metastasis-associated lncRNAs in breast cancer cells. Herein, we report a novel functional lncRNA, *LOC550643*, which is highly expressed in patients with breast cancer. In addition, high *LOC550643* expression levels are significantly correlated with poor survival in breast cancer patients. *LOC550643* is located on chromosome X (p11.21), and three alternative isoforms can be generated in breast cancer cells. Relevant studies have indicated that a known lncRNA termed *XIST* (X inactive specific transcript), which is located in the X inactivation center, and its product are transcribed from the inactive X chromosome (Yang et al., 2017). *XIST* is one of the first known examples of lncRNA with a prominent role in regulating X chromosome inactivation. In mammals, the majority of genes are silenced in one of the X-chromosomes in each cell, accounting for a similar level of gene expression between the sexes (Dey et al., 2014). *XIST* is typically expressed by all female somatic cells but not in female breast, ovarian, or cervical cancer cell lines (Kawakami et al., 2004; Benoît et al., 2007). *XIST* also plays a crucial role in ovarian cancer (Ren et al., 2015), non–small cell lung cancer (Tantai et al., 2015), and glioblastoma (Yao et al., 2015).

Zhu et al. (2015) first discovered *LOC550643* as a novel non-coding RNA mapped to Xp11.21. They observed that *LOC550643* was a susceptibility locus for systemic lupus erythematosus (SLE) and that it played a critical role in X-linked genetic variants in the pathogenesis of SLE in Han Chinese populations (Zhu et al., 2015). In order to avoid abnormal overexpression of X-linked genes, epigenetic modification participates to X chromosome inactivation in female cells (Brooks and Renaudineau, 2015). Zhu et al. (2015) hypothesized that *LOC550643* may maintain the X inactivation that prevents X-inked gene overexpression through dosage compensation in women. However additional studies are needed to determine how this locus influences the etiology of SLE (Zhu et al., 2015). Yang et al. (2017) discovered that *LOC550643* was highly expressed in nasopharyngeal carcinoma (NPC) and greatly associated with distant metastasis in patients with NPC. The researchers also observed that male patients with NPC had a higher tendency to express *LOC550643* than did female patients with NPC. Yang et al. (2017) suggested that high expression levels of *LOC550643* may act as a crucial role in NPC progression and its expression levels may use as a potential prognostic biomarker in NPC patients. Zhai et al. (2020) reported that high *LOC550643* expression contributed to pancreatic cancer progression by enhancing *KRAS* expression. Furthermore, *LOC550643* knockdown could suppress pancreatic cancer cell growth and invasion capability through releasing miR-494-3p sponging. Relevant studies have also investigated the role of *LOC550643* in thyroid cancer, revealing significantly higher levels of *LOC550643* expression in thyroid cancer cells and that *LOC550643* knockdown inhibited cancer cell growth by impairing cell cycle progression (Luo et al., 2020). By contrast, Konina et al. (2019) reported an opposite role, in which *LOC550643* inhibited melanoma cell migration ability but did not influence cell growth. An increasing number of studies have revealed that *LOC550643* is frequently overexpressed in human cancer cells and plays an oncogenic role in modulating cancer growth and metastasis. However, *LOC550643* expression might also suppress tumors in different cancer types, such as melanoma.

Our data indicate that *LOC550643* knockdown could suppress breast cancer cell proliferation by inducing cell cycle arrest in the S phase. The S phase (synthesis phase) is the part of the cell cycle in which DNA is replicated. The central machines that promote cell cycle progression are cyclin-dependent kinases (CDKs). Cyclin-binding enables inactive CDKs to become active (Barnum and O’Connell, 2014). Two types of cyclin are crucial for mitosis in animal cells: cyclin A, which is known mainly for its role in the S phase, in which it has been implicated in DNA replication, and cyclin B (De Boer et al., 2008; Gavet and Pines, 2010). In eukaryotes, cyclin B/CDK1 is essential for both entry and progression through mitosis (Gavet and Pines, 2010). Cyclin A/CDK2 expression is elevated at the onset of the S phase, and further activation occurs early in the G2 phase to regulate G2 phase progression (De Boer et al., 2008). CDK2 is considered a key CDK in the control of DNA replication in the S phase, even though CDK1 can compensate if CDK2 is disabled (Santamaría et al., 2007). Our data indicate that *LOC550643* knockdown resulted in lower cyclin B1, cyclin A2, and CDK2 protein levels in MDA-MB-231-IV2-1 cells and inhibited cell growth via promoting cell cycle arrest at the S phase. In addition, the current study indicated that *LOC550643* can sponge miR-125b-2-3p through direct interaction in breast cancer cells. Through a bioinformatics analysis, Shi et al. (2018) suggested that cyclin A2 is a potential target of miR-125b-2-3p. However, few studies have reported that miR-125b-2-3p contributes to cancer progression. Murray et al. (2014) revealed that serum levels of miR-125a-3p and miR-125b-2-3p increased before chemotherapy and subsequently decreased early following treatment in patients with pleuropulmonary blastoma (PPB). According to these results, miR-125b-2-3p may be useful as a serum biomarker for the early detection of PPB in patients with known germline *DICER1* mutations and for potential disease monitoring in patients with PPB (Murray et al., 2014). In addition, Lu et al. (2018) reported that miR-125b-2-3p sensitized colorectal cancer (CRC) to first-line chemotherapeutic therapy. Furthermore, the two-miRNA-based signatures (miR-125b-2-3p and miR-933) were a good prognostic and predictive biomarkers for tumor progression in advanced CRC, and they could be useful for providing standard first-line chemotherapy to patients with CRC (Lu et al., 2018). Meng et al. (2020) reported that high miR-125b-2-3p expression was correlated with metastasis and poor survival in patients with clear cell renal cell carcinoma. However, the biological function of miR-125b-2-3p in human cancer cells remains unknown. We identified that miR-125b-2-3p can suppress cell proliferation, colony formation, migration, and invasion capability in human breast cancer cells.

In summary, we identified a novel lncRNA, *LOC550643*, which acts as an oncogene in promoting breast cancer cell growth and metastasis. We also revealed that *LOC550643* expression can serve as a useful molecular biomarker for cancer diagnosis and as a potential therapeutic target for patients with breast cancer.

## Data Availability Statement

The original contributions presented in the study are publicly available. This data can be found here: https://www.ncbi.nlm.nih.gov/geo/, accession numbers: GSE175513 and GSE175514.

## Ethics Statement

The studies involving human participants were reviewed and approved by the Institutional Review Board of the Taipei Tzu Chi Hospital. Written informed consent for participation was not required for this study in accordance with the national legislation and the institutional requirements. The animal study was reviewed and approved by Animal Center and the Use Committee of Kaohsiung Veterans General Hospital (No. VGHKS-2017-2020-A042).

## Author Contributions

K-WT, Y-JC, and S-HC executed this study and drafted the manuscript. Y-TT and M-CL performed the biological function assay for the gastric cancer cells. Y-JC, K-HC, and Y-RC assisted in collecting clinical samples and analysis. K-WT, Y-JC, and L-HW supervised the study and edited the manuscript.

## Conflict of Interest

The authors declare that the research was conducted in the absence of any commercial or financial relationships that could be construed as a potential conflict of interest.

## Footnotes

1 https://tcga-data.nci.nih.gov/tcga/dataAccessMatrix.htm

## Supplementary Material

Supplementary Figure 1 Comparison of MDA-MB-231-P and MDA-MB-231-IV2-1 transcriptome profiles. **(A)** Results of differentially expressed protein-coding genes with twofold change (2926 upregulated and 2989 downregulated) in MDA-MB-231-IV2-1 cells compared with MDA-MB-231-P cells. **(B)** Scatter plot of total gene expressions in MDA-MB-231-P versus MDA-MB-231-IV2-1. **(C)** Gene ontology analysis of the twofold change in the protein-coding gene list revealing a significant enrichment in cell motility–related function.

Supplementary Figure 2 Kaplan–Meier analysis of the 52 metastasis-associated lncRNAs from TCGA data. Upper panel indicates the hazard ratio, and the lower panel is the log^(*p**v**a**l**u**e*)^. The log^(^*^*p*^* ^*value)*^ of <–1.3 is significant and marked in red (*p* < 0.05).

Supplementary Figure 3 Identification of the *LOC550643* gene structure in breast cancer cells by using RACE. **(A)** Genomic location of the *LOC550643* in the UCSC human genome database (upper panels). EST transcripts are in the lower panel. **(B)** Identification of the full length of *LOC550643* through 3′ and 5′ RACE. **(C)** Three transcripts of alternative splicing variants. **(D)** Three *LOC550643* isoforms aligned using a multiple alignment tool (http://www.ebi.ac.uk/Tools/msa/clustalo/).

Supplementary Figure 4 Expression levels and knockdown efficiency of *LOC550643* assessed in human breast cancer cells. **(A)** Expression levels of *LOC550643* assessed in human breast cancer cell lines by real-time PCR. **(B)** Schema of interaction sites of the two siRNA (si-*LOC550643*#301 and si-*LOC550643*#543) sequences designed for *LOC550643* knockdown. **(C)** After 48 h of individual siRNA of transfection (si-*LOC550643*#301, si-*LOC550643*#543, and N.C control) into MDA-MB-231-IV2-1 cells, *LOC550643* expression levels were examined using real-time PCR. Mock indicated a non-transfection control. The efficiency of si-*LOC550643*#301, si-*LOC550643*#543 *LOC550643* knockdown and mock was evaluated through comparisons with the control group.

Supplementary Figure 5 Expression levels of miRNA candidates assessed in human breast cancer cells from TCGA database. The expression levels of **(A)** miR-29b-5p, **(B)** miR-34b-3p, **(C)** miR-125b-2-3p, and **(D)** miR-629-3p were assessed in breast cancer tissue samples (*n* = 778) compared with adjacent normal tissue samples (*n* = 87; TCGA database).

Supplementary Figure 6 **(A)** Impacts of miR-125b-2-3p expression on the OS of patients with breast cancer. **(B)** Combination of *LOC550643* and miR-125b-2-3p expression levels was correlated with the survival curve of patients with breast cancer.
