# Supplementary figures and images for: LOC550643, a Long Non-coding RNA, Acts as Novel Oncogene in Regulating Breast Cancer Growth and Metastasis
Source: Front Cell Dev Biol. 2021 Jul 20;9:695632. doi: 10.3389/fcell.2021.695632 (PMC8329494; doi:10.3389/fcell.2021.695632)

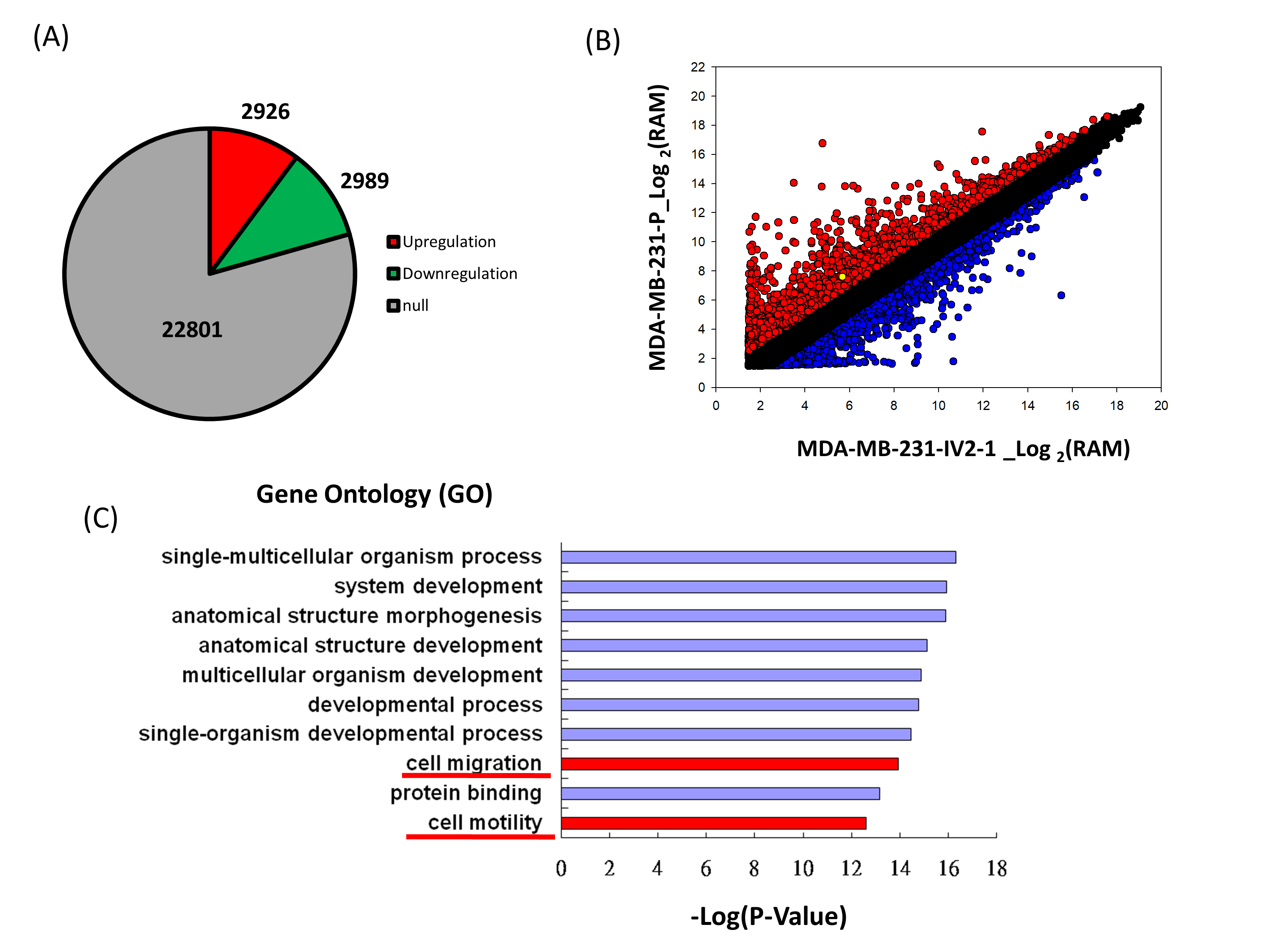

Supplement: Supplementary Figure 1 — Comparison of MDA-MB-231-P and MDA-MB-231-IV2-1 transcriptome profiles. (A) Results of differentially expressed protein-coding genes with twofold change (2926 upregulated and 2989 downregulated) in MDA-MB-231-IV2-1 cells compared with MDA-MB-231-P cells. (B) Scatter plot of total gene expressions in MDA-MB-231-P versus MDA-MB-231-IV2-1. (C) Gene ontology analysis of the twofold change in the protein-coding gene list revealing a significant enrichment in cell motility–related function. [file Image_1.TIF]

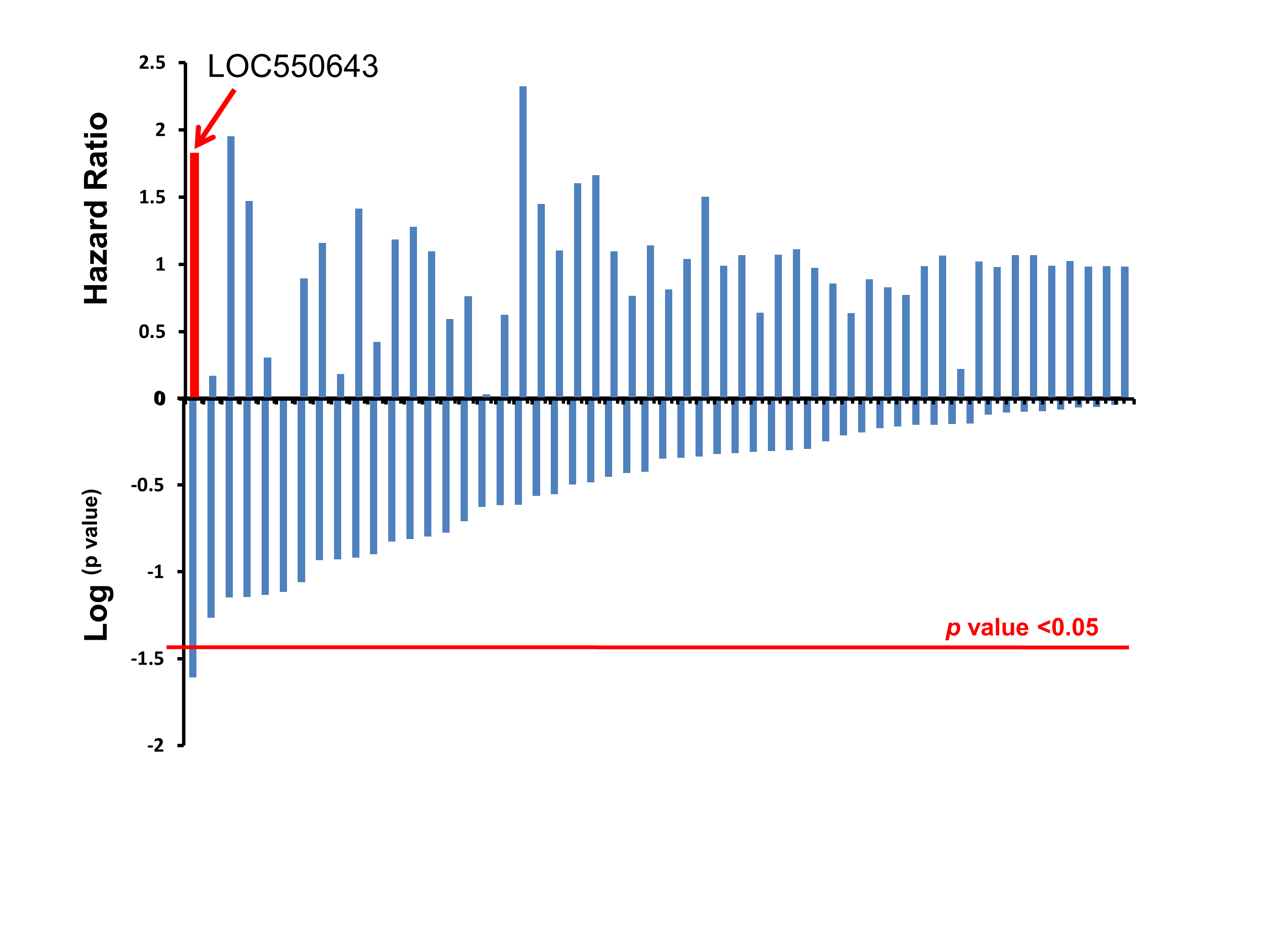

Supplement: Supplementary Figure 2 — Kaplan–Meier analysis of the 52 metastasis-associated lncRNAs from TCGA data. Upper panel indicates the hazard ratio, and the lower panel is the log(pvalue). The log(p value) of <–1.3 is significant and marked in red (p < 0.05). [file Image_2.TIF]

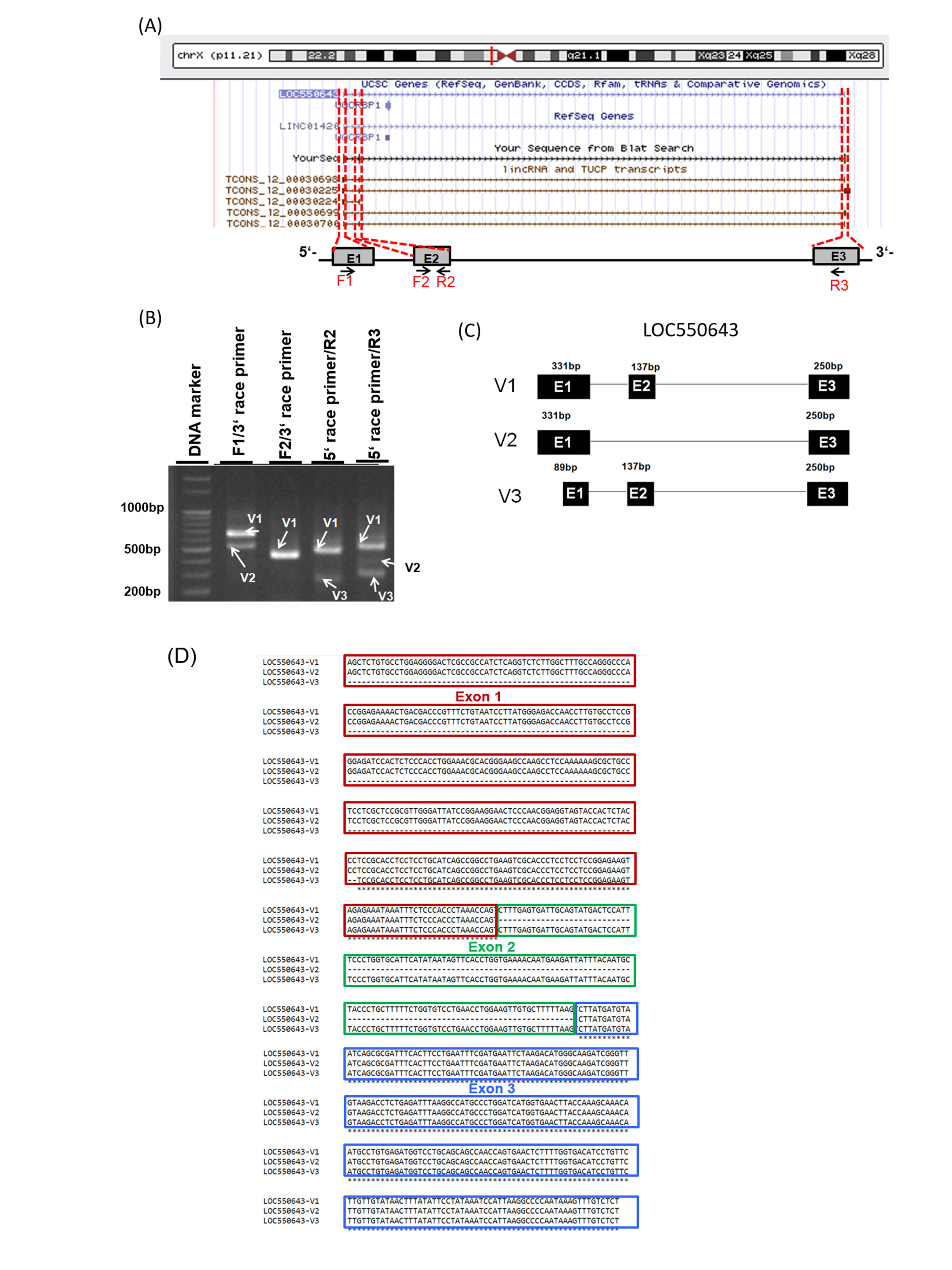

Supplement: Supplementary Figure 3 — Identification of the LOC550643 gene structure in breast cancer cells by using RACE. (A) Genomic location of the LOC550643 in the UCSC human genome database (upper panels). EST transcripts are in the lower panel. (B) Identification of the full length of LOC550643 through 3′ and 5′ RACE. (C) Three transcripts of alternative splicing variants. (D) Three LOC550643 isoforms aligned using a multiple alignment tool (http://www.ebi.ac.uk/Tools/msa/clustalo/). [file Image_3.TIF]

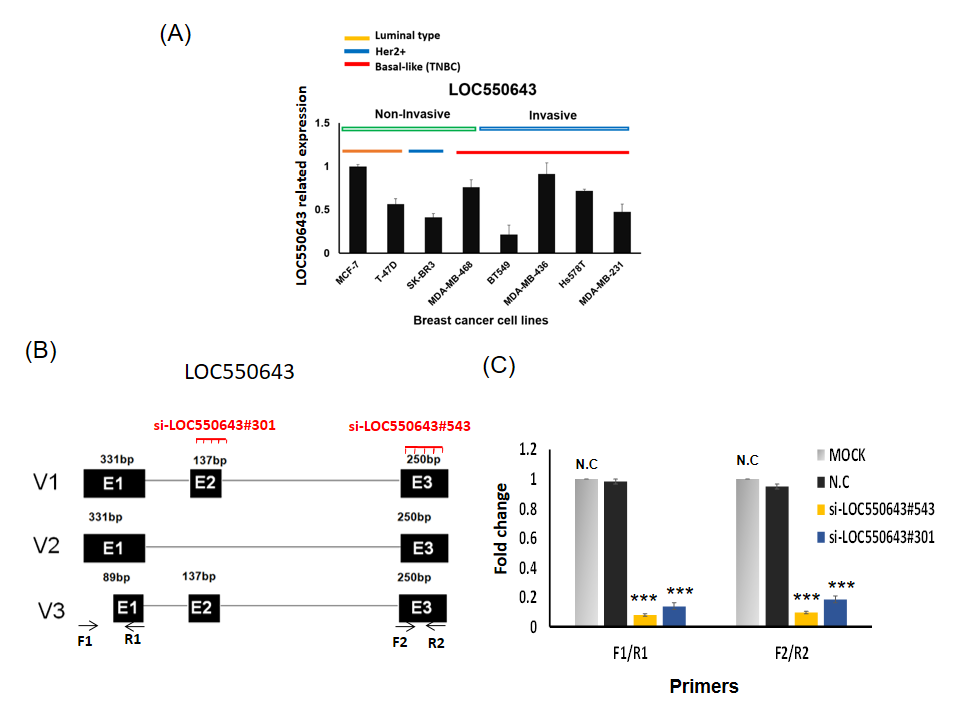

Supplement: Supplementary Figure 4 — Expression levels and knockdown efficiency of LOC550643 assessed in human breast cancer cells. (A) Expression levels of LOC550643 assessed in human breast cancer cell lines by real-time PCR. (B) Schema of interaction sites of the two siRNA (si-LOC550643#301 and si-LOC550643#543) sequences designed for LOC550643 knockdown. (C) After 48 h of individual siRNA of transfection (si-LOC550643#301, si-LOC550643#543, and N.C control) into MDA-MB-231-IV2-1 cells, LOC550643 expression levels were examined using real-time PCR. Mock indicated a non-transfection control. The efficiency of si-LOC550643#301, si-LOC550643#543 LOC550643 knockdown and mock was evaluated through comparisons with the control group. [file Image_4.TIF]

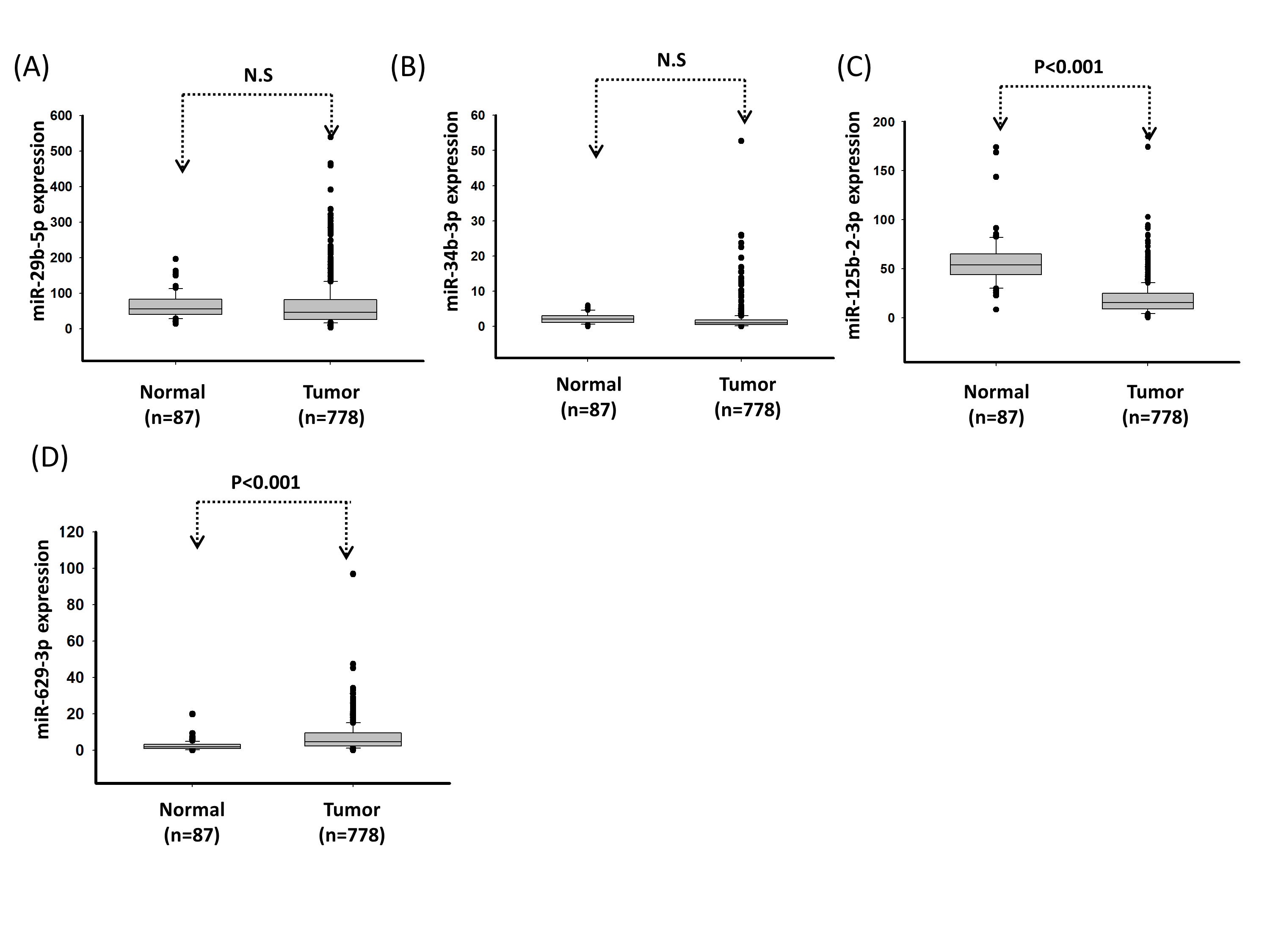

Supplement: Supplementary Figure 5 — Expression levels of miRNA candidates assessed in human breast cancer cells from TCGA database. The expression levels of (A) miR-29b-5p, (B) miR-34b-3p, (C) miR-125b-2-3p, and (D) miR-629-3p were assessed in breast cancer tissue samples (n = 778) compared with adjacent normal tissue samples (n = 87; TCGA database). [file Image_5.TIF]

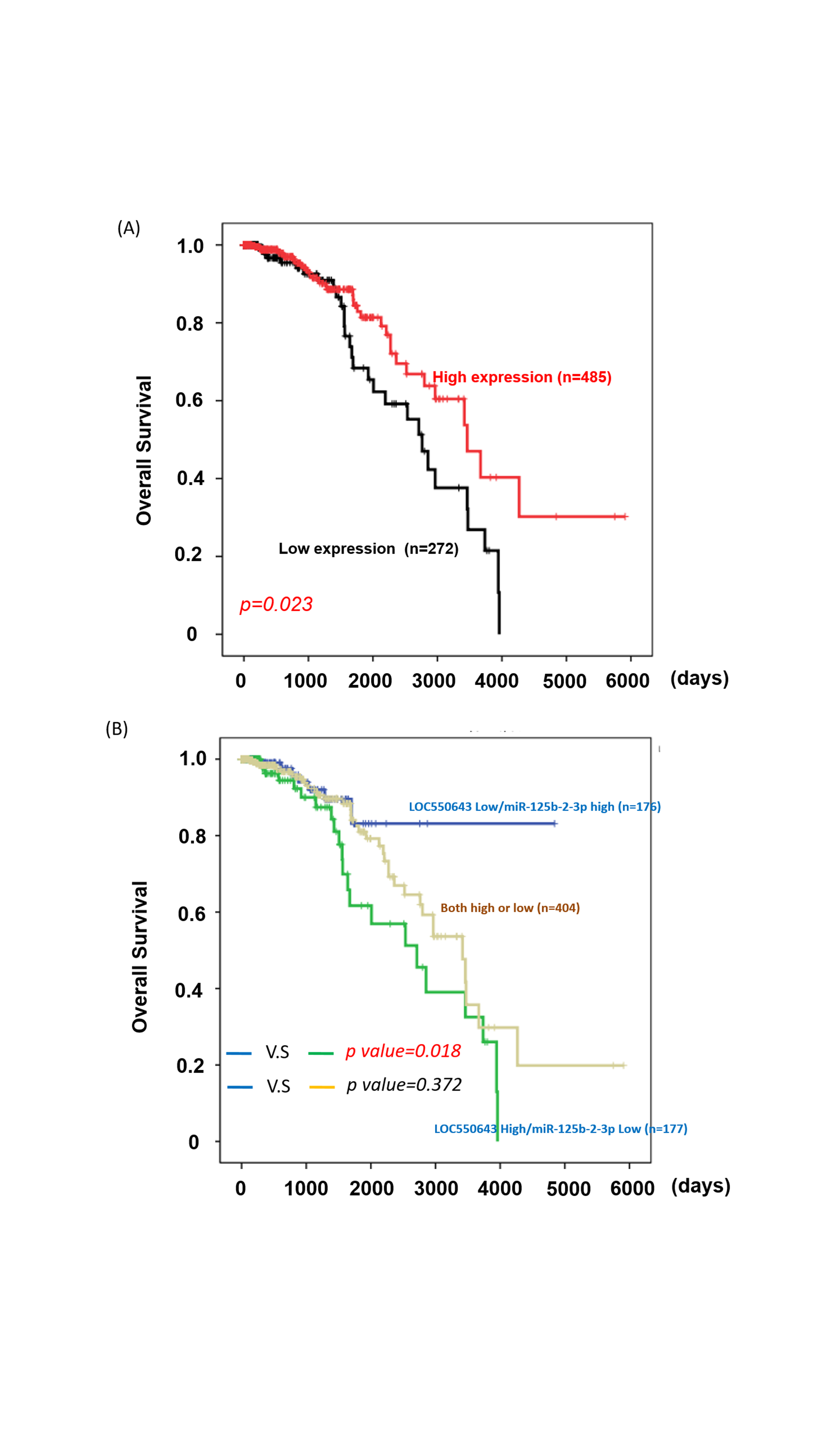

Supplement: Supplementary Figure 6 — (A) Impacts of miR-125b-2-3p expression on the OS of patients with breast cancer. (B) Combination of LOC550643 and miR-125b-2-3p expression levels was correlated with the survival curve of patients with breast cancer. [file Image_6.TIF]
