# Supplementary material for: LOC550643, a Long Non-coding RNA, Acts as Novel Oncogene in Regulating Breast Cancer Growth and Metastasis
Source: Front Cell Dev Biol. 2021 Jul 20;9:695632. doi: 10.3389/fcell.2021.695632 (PMC8329494; doi:10.3389/fcell.2021.695632)
Supplement: Supplementary file 7 [file Table_1.DOC]

|  | |
| --- | --- |
| **Primer** | **5'-3'** |
| GAPDH-F | TGCACCACCAACTGCTTAGC |
| GAPDH-R | GGCATGGACTGTGGTCATGAG |
| LOC652276-F | ATCCGTCTTGAAGGCAGCTC |
| LOC652276-R | CGTGGGCTTCAGTCACTACA |
| LOC550643-F | CCACCGGAGAAAACTGACGA |
| LOC550643-R | CCTCCGTTGGGAGTTCCTTC |
| PVT1-F | GGGGAATAACGCTGGTGGAA |
| PVT1-R | CCCATGGACATCCAAGCTGT |
| OR2A9P-F | TCAACCCGATGCTAAACCCC |
| OR2A9P-R | TTGGGCATCAAGAGTCCACG |
| RPL23AP53-F | AGATTCCAACGCAGCTTCA |
| RPL23AP53-R | CTCTGGCAACAGGGGTCTTT |
| GATs-F | CTCCAGCTCCCAGCCTATTG |
| GATs-R | GACACAGAACCTGTTGCTCG |
| U6-F | CTCGCTTCGGCAGCACA |
| U6-R | AACGCTTCACGAATTTGCGT |
| miR-125b-2-3p-RT | CTCAACTGGTGTCGTGGAGTCGGCAATTCAGTTGAGGTCCCAAG |
| miR-125b-2-3p-GSF | CGGCGGTCACAAGTCAGGCTCT |
| Universal-R | CTGGTGTCGTGGAGTCGGCAATTC |
| RACE-LOC550643-F1 | GGAGATCCACTCTCCCACCTGGAAA |
| RACE- LOC550643-F2 | GACTCCATTTCCCTGGTGCATTCA |
| RACE- LOC550643-R2 | CCAGGTTCAGGACACCAGAAAAAGCA |
| RACE- LOC550643-R3 | CAGGAAGTGAAATCGCGCTGATTACA |

**Supplementary Table 1. The Primers used in this study of qPCR and RLM-RACE**
