# Supplementary material for: LOC550643, a Long Non-coding RNA, Acts as Novel Oncogene in Regulating Breast Cancer Growth and Metastasis
Source: Front Cell Dev Biol. 2021 Jul 20;9:695632. doi: 10.3389/fcell.2021.695632 (PMC8329494; doi:10.3389/fcell.2021.695632)
Supplement: Supplementary file 8 [file Table_2.DOC]

| **Supplementary Table 2. The siRNA sequence used in this study** | |
| --- | --- |
| **siRNA** | **Sequence (5'-3')** |
| Negative control (N.C) | GCGACGAUCUGCCUAAGA |
| si-LOC550643#301 | CAGUCUUUGAGUGAUUGC |
| si-LOC550643#543 | CCUGGAUCAUGGUGAACU |
