# Supplementary material for: LOC550643, a Long Non-coding RNA, Acts as Novel Oncogene in Regulating Breast Cancer Growth and Metastasis
Source: Front Cell Dev Biol. 2021 Jul 20;9:695632. doi: 10.3389/fcell.2021.695632 (PMC8329494; doi:10.3389/fcell.2021.695632)
Supplement: Supplementary file 9 [file Table_3.doc]

| **Supplementary Table 3. The antibodies used in this study** | | | | | |
| --- | --- | --- | --- | --- | --- |
| **Antibody name** | **1st Ab Dilution** | **MW (kDa)** | **Company** | **Host** | **2nd Ab Dilution** |
| CDK1 | 1:200 | 34, 27 | 10762-1-AP, proteintech | Rabbit | 1:5000 |
| CDK2 | 1:1000 | 34 | MA5-17052, ThermoFisher | Mouse | 1:5000 |
| CDK4 | 1:1000 | 34 | MS-299-P, ThermoFisher | Mouse | 1:5000 |
| CyclinA2 | 1:500 | 56 | 18202-1-AP, proteintech | Rabbit | 1:5000 |
| CyclinB1 | 1:500 | 55-60 | 55004-1-AP, proteintech | Rabbit | 1:5000 |
| CyclinD1 | 1:200 | 36 | RM9104S, ThermoFisher | Rabbit | 1:5000 |
| P21 | 1:1000 | 21 | #2947, Cell signaling Technology, Inc., USA | Rabbit | 1:5000 |
| P27 | 1:500 | 27 | 25614-1-AP, proteintech | Rabbit | 1:5000 |
| β-actin (ACTB) | 1:5000 | 43 | MAB1501, Millipore | Mouse | 1:5000 |
| *MW: Molecular Weight | |  |  |  |  |
