# Supplementary material for: LOC550643, a Long Non-coding RNA, Acts as Novel Oncogene in Regulating Breast Cancer Growth and Metastasis
Source: Front Cell Dev Biol. 2021 Jul 20;9:695632. doi: 10.3389/fcell.2021.695632 (PMC8329494; doi:10.3389/fcell.2021.695632)
Supplement: Supplementary file 10 [file Table_4.DOC]

| Isoforms | Sequence |
| --- | --- |
| V1  (718bp) | AGCTCTGTGCCTGGAGGGGACTCGCCGCCATCTCAGGTCTCTTGGCTTTGCCAGGGCCCACCGGAGAAAACTGACGACCCGTTTCTGTAATCCTTATGGGAGACCAACCTTGTGCCTCCGGGAGATCCACTCTCCCACCTGGAAACGCACGGGAAGCCAAGCCTCCAAAAAAGCGCTGCCTCCTCGCTCCGCGTTGGGATTATCCGGAAGGAACTCCCAACGGAGGTAGTACCACTCTACCCTCCGCACCTCCTCCTGCATCAGCCGGCCTGAAGTCGCACCCTCCTCCTCCGGAGAAGTAGAGAAATAAATTTCTCCCACCCTAAACCAGTCTTTGAGTGATTGCAGTATGACTCCATTTCCCTGGTGCATTCATATAATAGTTCACCTGGTGAAAACAATGAAGATTATTTACAATGCTACCCTGCTTTTTCTGGTGTCCTGAACCTGGAAGTTGTGCTTTTTAAGTCTTATGATGTAATCAGCGCGATTTCACTTCCTGAATTTCGATGAATTCTAAGACATGGGCAAGATCGGGTTGTAAGACCTCTGAGATTTAAGGCCATGCCCTGGATCATGGTGAACTTACCAAAGCAAACAATGCCTGTGAGATGGTCCTGCAGCAGCCAACCAGTGAACTCTTTTGGTGACATCCTGTTCTTGTTGTATAACTTTATATTCCTATAAATCCATTAAGGCCCCAATAAAGTTTGTCTCT |
| V2  (581bp) | AGCTCTGTGCCTGGAGGGGACTCGCCGCCATCTCAGGTCTCTTGGCTTTGCCAGGGCCCACCGGAGAAAACTGACGACCCGTTTCTGTAATCCTTATGGGAGACCAACCTTGTGCCTCCGGGAGATCCACTCTCCCACCTGGAAACGCACGGGAAGCCAAGCCTCCAAAAAAGCGCTGCCTCCTCGCTCCGCGTTGGGATTATCCGGAAGGAACTCCCAACGGAGGTAGTACCACTCTACCCTCCGCACCTCCTCCTGCATCAGCCGGCCTGAAGTCGCACCCTCCTCCTCCGGAGAAGTAGAGAAATAAATTTCTCCCACCCTAAACCAGTCTTATGATGTAATCAGCGCGATTTCACTTCCTGAATTTCGATGAATTCTAAGACATGGGCAAGATCGGGTTGTAAGACCTCTGAGATTTAAGGCCATGCCCTGGATCATGGTGAACTTACCAAAGCAAACAATGCCTGTGAGATGGTCCTGCAGCAGCCAACCAGTGAACTCTTTTGGTGACATCCTGTTCTTGTTGTATAACTTTATATTCCTATAAATCCATTAAGGCCCCAATAAAGTTTGTCTCT |
| V3  (476bp) | TCCGCACCTCCTCCTGCATCAGCCGGCCTGAAGTCGCACCCTCCTCCTCCGGAGAAGTAGAGAAATAAATTTCTCCCACCCTAAACCAGTCTTTGAGTGATTGCAGTATGACTCCATTTCCCTGGTGCATTCATATAATAGTTCACCTGGTGAAAACAATGAAGATTATTTACAATGCTACCCTGCTTTTTCTGGTGTCCTGAACCTGGAAGTTGTGCTTTTTAAGTCTTATGATGTAATCAGCGCGATTTCACTTCCTGAATTTCGATGAATTCTAAGACATGGGCAAGATCGGGTTGTAAGACCTCTGAGATTTAAGGCCATGCCCTGGATCATGGTGAACTTACCAAAGCAAACAATGCCTGTGAGATGGTCCTGCAGCAGCCAACCAGTGAACTCTTTTGGTGACATCCTGTTCTTGTTGTATAACTTTATATTCCTATAAATCCATTAAGGCCCCAATAAAGTTTGTCTCT |

**Supplementary Table 4. The sequence of three isoforms of LOC550643 was examined by Sanger sequencing**
